# Enhancing enterocyte fatty acid oxidation in mice affects glycemic control depending on dietary fat

**DOI:** 10.1038/s41598-018-29139-6

**Published:** 2018-07-17

**Authors:** Deepti Ramachandran, Rosmarie Clara, Shahana Fedele, Ladina Michel, Johannes Burkard, Sharon Kaufman, Abdiel Alvarado Diaz, Nadja Weissfeld, Katrien De Bock, Carina Prip-Buus, Wolfgang Langhans, Abdelhak Mansouri

**Affiliations:** 1 Physiology and Behavior Laboratory, ETH Zurich, Schwerzenbach, Switzerland; 2 Excercise and Health Laboratory, ETH Zurich, Schwerzenbach, Switzerland; 3 Inserm, U1016, Institut Cochin, Paris, France; 4 CNRS, UMR, 8104 Paris, France; 5 Université Paris Descartes, Sorbonne Paris Cité, Paris, France

## Abstract

Studies indicate that modulating enterocyte metabolism might affect whole body glucose homeostasis and the development of diet-induced obesity (DIO). We tested whether enhancing enterocyte fatty acid oxidation (FAO) could protect mice from DIO and impaired glycemic control. To this end, we used mice expressing a mutant form of carnitine palmitoyltransferase-1a (CPT1mt), insensitive to inhibition by malonyl-CoA, in their enterocytes (iCPT1mt) and fed them low-fat control diet (CD) or high-fat diet (HFD) chronically. CPT1mt expression led to an upregulation of FAO in the enterocytes. On CD, iCPT1mt mice had impaired glycemic control and showed concomitant activation of lipogenesis, glycolysis and gluconeogenesis in their enterocytes. On HFD, both iCPT1mt and control mice developed DIO, but iCPT1mt mice showed improved glycemic control and reduced visceral fat mass. Together these data indicate that modulating enterocyte metabolism in iCPT1mt mice affects glycemic control in a body weight-independent, but dietary fat-dependent manner.

## Introduction

Obesity and its related comorbidities, such as type-2-diabetes (T2D), hypertension, and cardiovascular disease, are major global health concerns^1^. With a rise in the consumption of western diets, and with decreasing physical activity, the incidence of obesity is increasing world over. Currently, the only treatments for morbid obesity that lead to sustained weight loss are invasive and costly interventions such as gastric bypass surgery^2^. The success of these surgical procedures comes with several unresolved side effects, including malabsorption of essential micronutrients and early or late post-surgical complications^2^. Interestingly, one of the consistent benefits of bariatric surgeries is improved glycemic control based on a reversal of insulin resistance (IR). These improvements are seen even before any noticeable weight loss^3^. Results from gastric bypass rodent models as well as human patients suggest that these almost immediate improvements are due to functional and/or morphological changes in the small intestine^4,5^.

Previous pharmacological studies in rodents implicated enhanced fatty acid oxidation (FAO) in the small intestine in the control of eating^6–8^. We recently reported evidence that a constitutive overexpression of the mitochondrial protein Sirtuin 3 (SIRT3) in mouse enterocytes was associated with enhanced FAO and ketogenesis and reduced fatty acid synthesis in these cells when the mice were fed a fat-rich diet^9^. Interestingly, constitutive enterocyte SIRT3 overexpression had no effect on daily food intake and did not protect the mice from developing diet-induced obesity (DIO), but did protect them from developing IR. SIRT3, however, is a post-translational regulator of several other pathways in addition to FAO, including reactive oxygen species (ROS) scavenging, the tri-carboxylic acid (TCA) cycle, urea cycle and ketogenesis^10^.

To more specifically upregulate FAO in mouse enterocytes, we used the CPT1mt protein, a mutated form of the rat carnitine palmitoyltransferase-1a (CPT1a) enzyme that is insensitive to its endogenous inhibitor malonyl-CoA^11^. Several studies have used the CPT1mt protein *in vitro* as well as *in vivo* to enhance mitochondrial FAO flux in target cells or tissues^12–17^. We crossed the established transgenic mouse line with a floxed STOP cassette preceding the Cpt1mt gene (Cpt1mt^fl/fl^)^17^ with the Villin-Cre mouse line (Vil-Cre^+/−^)^18^ to generate mice with a homozygous expression of CPT1mt in the enterocytes (iCPT1mt). We isolated primary enterocytes from the duodenum and jejunum of these mice to test the metabolic flux of these cells. We fed iCPT1mt and Cpt1mt^fl/fl^ mice a low-fat control diet (CD) or a high-fat diet (HFD) for several weeks, and phenotyped them to determine whether enterocyte CPT1mt expression could protect these mice from developing impaired glucose homeostasis, IR and DIO.

## Materials and Methods

### Animals

Transgenic mice on the C57Bl6N background homozygous for the Cpt1mt construct, *lox*P - STOP cassette - *lox*P - Cpt1mt (Cptmt^fl/fl^)^17^ were crossed with mice on a C57Bl6J background with hemizygous expression of Cre recombinase under the Villin promoter (VilCre^+/−^)^18^ to generate mice hemizygous for both the Cpt1mt gene and the Villin-Cre gene (Cptmt^fl/-^/ VilCre^+/−^). These mice were backcrossed with the parental line Cptm^fl/fl^ to generate male mice homozygous for the Cpt1mt floxed cassette and hemizygous for Villin-Cre (Cptmt^fl/fl^/VilCre^+/−^ or iCPT1mt) expressing CPT1mt specifically in the epithelial cells of the intestine. The Cptmt^fl/fl^ male littermates served as controls. All mice were genotyped immediately after weaning (at 3 to 4 weeks of age) and recaged in groups (2–4 mice/cage) such that only mice with the same genotype shared cages. All breedings were carried out in our in-house specified and opportunistic pathogen free (SOPF) facility. At 10 to 12 weeks of age, mice were moved into the experimental room with controlled temperature and humidity (22 ± 1 °C, 55 ± 5%) and a reversed 12 h/12 h dark/light cycle (lights off at 8 am). Animals had *ad libitum* access to food and water unless otherwise specified. All animal experimental protocols were performed in accordance with the Swiss animal welfare legislation, and approved by the Cantonal Veterinary Office of Zurich.

### Diet

Mice in the SOPF breeding facility were fed autoclaved chow diet (#3807, Kliba). After 1–2 weeks of adaptation to experimental room conditions, all experimental mice were fed either standard chow (#3430, Kliba), refined control diet (CD, #S9213-E001, 10% of energy from fat) or high-fat diet (HFD, #E15742-34, 60% of energy from fat) from Ssniff Spezialdiäten GmbH.

### Body weight measurements

Body weights of mice fed CD and HFD were monitored regularly in the dark phase as indicated using a generic weighing scale.

### Insulin sensitivity test (IST)

Mice were fasted for 5–6 h in the middle of the dark phase with *ad libitum* access to water. Actrapid HM human insulin (Novo Nordisk) was injected intraperitoneally (IP), and tail blood glucose was monitored at the indicated time points using the Accu-Chek Aviva blood glucose monitor (Roche). Insulin dose: 0.4 mU/g body weight (CD) and 0.8 mU/g body weight (HFD)^19^.

### Oral glucose tolerance test (OGTT)

After a 6 h fast from dark phase onset with *ad libitum* access to water^20^ mice received a 20% glucose solution by gavage (solvent: water, glucose dose: 2 g/kg body weight). Tail blood glucose was monitored at the time points indicated.

### Intraperitoneal glucose tolerance test (IPGTT)

After a 6 h fast from dark phase onset with ad libitum access to water^20^ mice were injected IP with a 20% glucose solution (solvent: 0.9% saline, glucose dose: 2 g/kg body weight). Tail blood glucose was monitored at the time points indicated.

### Body composition

Mice were scanned under isoflurane anesthesia using a high-resolution micro computed tomography (CT) scanner (La Theta LCT-100; Hitachi-Aloka Medical Ltd), to determine body composition and fat distribution.

### Indirect calorimetry

Measurements were carried out using the Phenomaster/Labmaster metabolic cages (TSE systems). Mice were adapted to single housing in cages similar to the Phenomaster cages for at least one week prior to measurements. Data displayed were collected after additional 2 days of habituation in the system.

### Animal sacrifice and tissue collection

Mice were food deprived for 2 h prior to sacrifice unless specified otherwise. All animals were sacrificed in the dark phase by decapitation, and trunk blood was collected in tubes containing 0.5 M EDTA. The blood samples were centrifuged at 8,700 g for 10 min at 4 °C, and plasma was collected and stored at −80 °C until required. The intestine and liver were dissected out. Intestinal samples were further processed as described below, and the liver was flash frozen in liquid nitrogen and stored at −80 °C until required. Enterocytes were isolated using a modified protocol described earlier^9,21^ using 12.5 mL distritip maxi syringes (#F164120, Gilson) and ice-cold Cell Recovery solution (#354253, Corning). The cells were scraped into ice cold PBS, pelleted and pellets were snap frozen in liquid nitrogen and stored at −80 °C until required.

### Primary enterocyte FAO assay

Fifteen to 20-week-old iCPT1mt and CPT1mt^fl/fl^ mice were fasted overnight. Enterocytes were isolated as described above and scraped into ice cold petri dishes containing DMEM (#A1443001, Gibco, ThermoFisher) supplemented with 100 U/mL penicillin and streptomycin (pen-strep), 1 × N-2 supplement, 1 × B-27 supplement, 10 µM Y-27632 and 2 µM N-acetyl cysteine (NAC) and phenol red (basal medium). These cells were then transferred to a 50 mL tube and centrifuged at 500 g for 6 min at room temperature. The cell pellet was resuspended in pre-warmed 0.25% trypsin-EDTA and incubated in a 37 °C, 5% CO_2_ incubator for 7 min. An equal volume of soybean trypsin inhibitor (#T6414, SIGMA) containing 20 µM Y-27632 was then added to neutralize trypsin activity. The cells were filtered successively through 100 µ and 40 µ filters. The filtrate was kept on ice, the cells were counted, and an appropriate number of cells was collected in a 2 mL Eppendorf tube, centrifuged at 500 g for 5 min and the resulting pellet was resuspended in ice cold basal medium. An equal volume of ice cold growth factor reduced Matrigel (#354230, Corning) diluted 1:2 in the same medium (to an effective concentration of approximately 5 mg/mL) was added to these cells so that the final concentration of matrigel in the cell suspension was 1:4 (2.5 mg/mL). 250000 (250 K) cells were plated per well in 40 µL of 1:4 matrigel in a regular 96 well cell culture plate. The plate was incubated on ice for 20 min for the cells to settle down through the viscous matrigel, then transferred to a 37 °C, 5% CO_2_ incubator for 20 min for the matrigel to solidify. Basal medium was further supplemented with 1.2 × glucose, L-carnitine, fatty acid free BSA, cold palmitic acid and hot (^3^H-9,10)-palmitic acid, now termed as FAO assay medium. Prewarmed FAO assay medium was added to each well (210 µL medium per well) such that each well now had a total volume of 250 µL of medium with a final (1×) concentration of 2.5 mM glucose, 500 µM L-carnitine, 50 µM fatty acid free BSA, 100 µM cold palmitic acid and 2 µCi/ml of hot (^3^H-9,10)-palmitic acid. The cells were incubated for 5 h in a 37 °C, 5% CO_2_ incubator. Subsequently, 200 µL of medium from each well was transferred into a glass vial and 50 µL of 3 M perchloric acid was added to each vial to stop any metabolic activity. Each vial was closed with a rubber stopper equipped with a hanging well containing a filter paper (1 × 6 cm; #3030-931, Whatman) soaked in 200 µL of water, and then the vials were incubated for 48 h at 37 °C. The filter paper was then carefully transferred into a scintillation vial containing 5 mL of Ultima Gold scintillation fluid (#6013329, Perkin Elmer) along with 100 µL of water used to wash any condensation in the hanging well. The disintegrations per minute were recorded using the 2000CA liquid scintillation analyzer (Tri-Carb). The FAO assay medium containing cold plus hot palmitic acid was used to generate a standard curve using which the FAO flux was calculated from the disintegrations per minute for each sample. Enterocytes from 3 animals were pooled for each genotype (biological replicates) and the n values in the figure legends refer to technical replicates.

### Primary enterocyte extracellular flux analysis

Primary enterocytes were subjected to metabolic flux analysis using the extracellular flux analyzer XFe96 (Agilent Seahorse XF technology) subsequently referred to as the “seahorse”. Enterocytes, from 15- to 20-week old iCPT1mt and CPT1mt^fl/fl^ mice fasted overnight, were isolated as described above and scraped into a petri dish containing DMEM (#A1443001, Gibco, ThermoFisher) supplemented with 5 mM glucose, 100 U/mL pen-strep, 1 × glutamax, 1 × sodium pyruvate, 1 × N-2 supplement, 1 × B-27 supplement, 10 µM Y-27632 (ROCK inhibitor) and 2 µM NAC. The dissociated and filtered cells were counted, centrifuged at 500 g for 6 min and an appropriate number of cells were resuspended in Krebs-Henseleit Buffer (KHB) containing 111 mM NaCl, 4.7 mM KCl, 1.25 mM CaCl_2_, 2 mM MgSO_4_, 1.2 mM NaH_2_PO_4_ supplemented with 100 U/mL pen-strep, 1 × N-2 supplement, 1 × B-27 supplement, 10 µM Y27632 and 2 µM NAC, pH 7.4) further referred to as experimental medium. The cells were plated into the 96 well seahorse cell plate kept on ice so that each well was seeded with 250 K cells in a volume of 20 µL of 1:4 matrigel diluted in experimental medium. The plate was incubated on ice for 20 min and in a 37 °C, 5% CO_2_ incubator for 20 min. Pre-warmed experimental medium (160 µL) was then added to each well. The cells were incubated in a CO_2_ free incubator at 37 °C for 30 min while the machine was calibrated. Basal oxygen consumption rate (OCR) and extracellular acidification rate (ECAR) were measured by the seahorse analyzer followed by the mitochondrial stress test using appropriate concentrations (see figure legends) of the respiratory poisons Oligomycin (Oligo), Carbonyl cyanide-4-phenylhydrazone (FCCP), Antimycin and Rotenone (Anti + Rot) as indicated. It should be noted that some wells showed a leak from one or more of the B, C or D ports of the cartridge due to capillary action initiated by the medium from the wells touching the ports of the cartridge during the mixing process. We believe that this occurred due to the consistency and thickness of the volume of matrigel in the measurement chamber. These wells were easily identifiable by their decrease in OCR due to oligo or Anti + Rot leak or an increase in OCR due to an FCCP leak even when no compounds were injected into the ports. These wells were eliminated from the analysis. For every parameter we used the average of the three time points measured after the relevant injection/treatment using the equations described below^22^.

$$\begin{array}{ccc}{\rm{N}}{\rm{o}}{\rm{n}} \mbox{-} {\rm{m}}{\rm{i}}{\rm{t}}{\rm{o}}{\rm{c}}{\rm{h}}{\rm{o}}{\rm{n}}{\rm{d}}{\rm{r}}{\rm{i}}{\rm{a}}{\rm{l}}\,{\rm{r}}{\rm{e}}{\rm{s}}{\rm{p}}{\rm{i}}{\rm{r}}{\rm{a}}{\rm{t}}{\rm{i}}{\rm{o}}{\rm{n}} & = & {\rm{A}}{\rm{v}}{\rm{e}}{\rm{r}}{\rm{a}}{\rm{g}}{\rm{e}}\,{\rm{o}}{\rm{f}}\,{\rm{t}}{\rm{h}}{\rm{r}}{\rm{e}}{\rm{e}}\,{\rm{O}}{\rm{C}}{\rm{R}}\,{\rm{m}}{\rm{e}}{\rm{a}}{\rm{s}}{\rm{u}}{\rm{r}}{\rm{e}}{\rm{m}}{\rm{e}}{\rm{n}}{\rm{t}}{\rm{s}}\,{\rm{a}}{\rm{f}}{\rm{t}}{\rm{e}}{\rm{r}}\,{\rm{A}}{\rm{n}}{\rm{t}}{\rm{i}}+{\rm{R}}{\rm{o}}{\rm{t}}\\ {\rm{B}}{\rm{a}}{\rm{s}}{\rm{a}}{\rm{l}}\,{\rm{O}}{\rm{C}}{\rm{R}} & = & {\rm{A}}{\rm{v}}{\rm{e}}{\rm{r}}{\rm{a}}{\rm{g}}{\rm{e}}\,{\rm{o}}{\rm{f}}\,{\rm{t}}{\rm{h}}{\rm{r}}{\rm{e}}{\rm{e}}\,{\rm{O}}{\rm{C}}{\rm{R}}\,{\rm{m}}{\rm{e}}{\rm{a}}{\rm{s}}{\rm{u}}{\rm{r}}{\rm{e}}{\rm{m}}{\rm{e}}{\rm{n}}{\rm{t}}{\rm{s}}\,{\rm{a}}{\rm{f}}{\rm{t}}{\rm{e}}{\rm{r}}\,{\rm{g}}{\rm{l}}{\rm{u}}{\rm{c}}{\rm{o}}{\rm{s}}{\rm{e}}\\  &  & -{\rm{N}}{\rm{o}}{\rm{n}} \mbox{-} {\rm{m}}{\rm{i}}{\rm{t}}{\rm{o}}{\rm{c}}{\rm{h}}{\rm{o}}{\rm{n}}{\rm{d}}{\rm{r}}{\rm{i}}{\rm{a}}{\rm{l}}\,{\rm{r}}{\rm{e}}{\rm{s}}{\rm{p}}{\rm{i}}{\rm{r}}{\rm{a}}{\rm{t}}{\rm{i}}{\rm{o}}{\rm{n}}\\ {\rm{M}}{\rm{a}}{\rm{x}}{\rm{i}}{\rm{m}}{\rm{a}}{\rm{l}}\,{\rm{r}}{\rm{e}}{\rm{s}}{\rm{p}}{\rm{i}}{\rm{r}}{\rm{a}}{\rm{t}}{\rm{i}}{\rm{o}}{\rm{n}} & = & {\rm{A}}{\rm{v}}{\rm{e}}{\rm{r}}{\rm{a}}{\rm{g}}{\rm{e}}\,{\rm{o}}{\rm{f}}\,{\rm{t}}{\rm{h}}{\rm{r}}{\rm{e}}{\rm{e}}\,{\rm{O}}{\rm{C}}{\rm{R}}\,{\rm{m}}{\rm{e}}{\rm{a}}{\rm{s}}{\rm{u}}{\rm{r}}{\rm{e}}{\rm{m}}{\rm{e}}{\rm{n}}{\rm{t}}{\rm{s}}\,{\rm{a}}{\rm{f}}{\rm{t}}{\rm{e}}{\rm{r}}\,{\rm{F}}{\rm{C}}{\rm{C}}{\rm{P}}\\  &  & -{\rm{N}}{\rm{o}}{\rm{n}} \mbox{-} {\rm{m}}{\rm{i}}{\rm{t}}{\rm{o}}{\rm{c}}{\rm{h}}{\rm{o}}{\rm{n}}{\rm{d}}{\rm{r}}{\rm{i}}{\rm{a}}{\rm{l}}\,{\rm{r}}{\rm{e}}{\rm{s}}{\rm{p}}{\rm{i}}{\rm{r}}{\rm{a}}{\rm{t}}{\rm{i}}{\rm{o}}{\rm{n}}\\ {\rm{P}}{\rm{r}}{\rm{o}}{\rm{t}}{\rm{o}}{\rm{n}}\,{\rm{l}}{\rm{e}}{\rm{a}}{\rm{k}} & = & {\rm{A}}{\rm{v}}{\rm{e}}{\rm{r}}{\rm{a}}{\rm{g}}{\rm{e}}\,{\rm{o}}{\rm{f}}\,{\rm{t}}{\rm{h}}{\rm{r}}{\rm{e}}{\rm{e}}\,{\rm{O}}{\rm{C}}{\rm{R}}\,{\rm{m}}{\rm{e}}{\rm{a}}{\rm{s}}{\rm{u}}{\rm{r}}{\rm{e}}{\rm{m}}{\rm{e}}{\rm{n}}{\rm{t}}{\rm{s}}\,{\rm{a}}{\rm{f}}{\rm{t}}{\rm{e}}{\rm{r}}\,{\rm{o}}{\rm{l}}{\rm{i}}{\rm{g}}{\rm{o}}{\rm{m}}{\rm{y}}{\rm{c}}{\rm{i}}{\rm{n}}\\  &  & -{\rm{N}}{\rm{o}}{\rm{n}} \mbox{-} {\rm{m}}{\rm{i}}{\rm{t}}{\rm{o}}{\rm{c}}{\rm{h}}{\rm{o}}{\rm{n}}{\rm{d}}{\rm{r}}{\rm{i}}{\rm{a}}{\rm{l}}\,{\rm{r}}{\rm{e}}{\rm{s}}{\rm{p}}{\rm{i}}{\rm{r}}{\rm{a}}{\rm{t}}{\rm{i}}{\rm{o}}{\rm{n}}\\ {\rm{R}}{\rm{e}}{\rm{s}}{\rm{p}}{\rm{i}}{\rm{r}}{\rm{a}}{\rm{t}}{\rm{i}}{\rm{o}}{\rm{n}}\,{\rm{l}}{\rm{i}}{\rm{n}}{\rm{k}}{\rm{e}}{\rm{d}}\,{\rm{t}}{\rm{o}}\,{\rm{A}}{\rm{T}}{\rm{P}}\,{\rm{s}}{\rm{y}}{\rm{n}}{\rm{t}}{\rm{h}}{\rm{e}}{\rm{s}}{\rm{i}}{\rm{s}} & = & {\rm{B}}{\rm{a}}{\rm{s}}{\rm{a}}{\rm{l}}\,{\rm{O}}{\rm{C}}{\rm{R}}-{\rm{P}}{\rm{r}}{\rm{o}}{\rm{t}}{\rm{o}}{\rm{n}}\,{\rm{l}}{\rm{e}}{\rm{a}}{\rm{k}}\\ {\rm{S}}{\rm{p}}{\rm{a}}{\rm{r}}{\rm{e}}\,{\rm{r}}{\rm{e}}{\rm{s}}{\rm{p}}{\rm{i}}{\rm{r}}{\rm{a}}{\rm{t}}{\rm{o}}{\rm{r}}{\rm{y}}\,{\rm{c}}{\rm{a}}{\rm{p}}{\rm{a}}{\rm{c}}{\rm{i}}{\rm{t}}{\rm{y}} & = & {\rm{M}}{\rm{a}}{\rm{x}}{\rm{i}}{\rm{m}}{\rm{a}}{\rm{l}}\,{\rm{r}}{\rm{e}}{\rm{s}}{\rm{p}}{\rm{i}}{\rm{r}}{\rm{a}}{\rm{t}}{\rm{i}}{\rm{o}}{\rm{n}}-{\rm{B}}{\rm{a}}{\rm{s}}{\rm{a}}{\rm{l}}\,{\rm{O}}{\rm{C}}{\rm{R}}\end{array}$$

Glycolysis, glycolytic capacity and glycolytic reserve were calculated from the glycolytic stress test using the averages of the three measurements made after the appropriate compound injection as described earlier^23^ as well as in the manufacturer’s protocol. In brief, glycolytic flux refers to the difference between the mean of measurements after glucose injection and baseline values. Glycolytic capacity refers to the difference between the mean measurements after oligo injection and baseline values, and glycolytic reserve refers to the difference between the mean measurements after oligo injection and after glucose injection. Each well represented a biological replicate of 1, while the n values in the figure legends refer to technical replicates.

### Western blotting

Tissue samples were processed for western blotting as described earlier^16^. Briefly, the samples were lysed in RIPA buffer, protein concentrations estimated and denatured in Laemmli buffer containing DTT. Equal amounts of protein samples were run in SDS-PAGE gels and blotted onto PVDF membranes and probed using primary antibodies for CPT1a^24^, HMGCS2 (#sc-33828, Santa Cruz Biotechnology) and β-actin (#A2228, Sigma) and the appropriate HRP-linked secondary antibody. The blots were developed using an in-house chemiluminescence-based detection kit and analyzed using the ImageQuant LAS 4000 mini (GE Health Care).

### Histology - H&E stain

Two cm pieces of the duodenum and jejunum, and 1 cm^3^ liver samples were collected and processed for paraffin embedding and H&E staining as described earlier^9^.

### RT-qPCR analysis

RNA was extracted from tissue samples using the Trizol reagent (#15596018, Life Technologies) following the manufacturer’s protocol and treated with DNAse (#79254, Quiagen). cDNA was synthesized using the High-Capacity cDNA Reverse Transcription Kit (#4368813, Applied Biosystems) and used for real time quantitative PCR (RT-qPCR) reactions using FAST SYBR green and the Viia7 Real Time PCR system (Applied Biosystems). Each sample was run in triplicate and the best duplicates or triplicates were analyzed using the 2^−∆∆C^_T_ method^25^ with Ppib as the reference gene^26^. Primers used are listed in Sup Table 1.

### Statistical analysis

All data were analyzed using GraphPad Prism (version 7.0.2) or IBM SPSS statistics (version 23). Outliers were identified using the Grubb’s test and removed. Data normality was verified using the Shapiro-Wilk test (when n ≥ 7) and Kolmogorov-Smirnov test (when 5 ≤ n ≤ 6). Parametric data was analyzed using an unpaired Student’s t-test (two-tailed) or 2 × 2 factorial parametric analysis of variance followed by Sidak’s multiple comparison *post hoc* test whenever appropriate or a mixed repeated measures ANOVA followed by a simple effects analysis in the case of a significant interaction or a univariate ANCOVA. The nonparametric test (Mann-Whitney) was used when the data did not meet the criteria of normal distribution. Statistical tests used and *P*-values are mentioned in the figure legends. *P*-values less than 0.05 were considered significant.

## Results

### iCPT1mt mice show increased CPT1a expression in the intestine, but not in the liver

**Figure 1:**
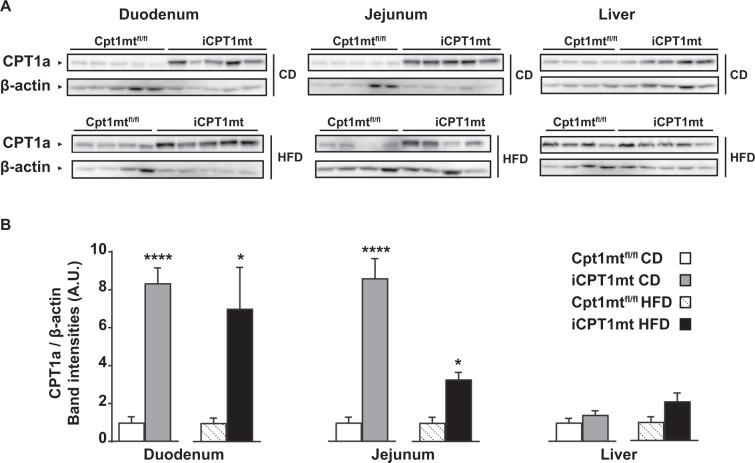
iCPTmt mice show increased CPT1a expression in the intestine, but not in the liver. Western blot analysis for carnitine palmitoyltransferase-1, liver isoform (CPT1a) and β-actin protein expression from tissue samples of Cpt1mt^fl/fl^ and iCPT1mt mice fed control diet (CD) or high-fat diet (HFD) for 20 weeks. (**A**) Western blot bands from the duodenum, jejunum and liver. (**B**) Quantification of band intensities. (n = 4 to 5, Unpaired t test; *P < 0.05 and ****P < 0.0001 for duodenum CD and HFD, jejunum CD and liver CD and HFD; Mann-Whitney test; *P < 0.05 for jejunum HFD). Data are presented as mean values ± SEM.

Western blot analysis of tissue samples from Cpt1mt^fl/fl^ and iCPT1mt mice fed CD and HFD indicated that the CPT1mt protein was expressed in the enterocytes of the duodenum and jejunum, but not in the liver of iCPT1mt mice (*P* < 0.0001 for CD duodenum and jejunum; *P* < 0.05 for HFD duodenum and jejunum) (Fig. 1A,B).

### Primary enterocytes from iCPT1mt mice have a higher rate of FAO, mitochondrial respiration and glycolysis than Cpt1mt^fl/fl^ control mice

**Figure 2:**
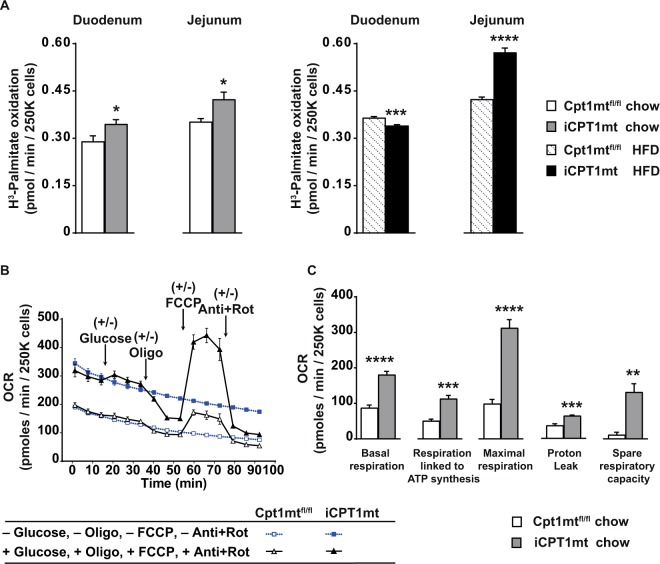
Primary enterocytes from iCPT1mt mice have a higher rate of FAO, mitochondrial respiration and glycolysis than Cpt1mt^fl/fl^ control mice. (**A**) Rate of oxidation of H^3^-Palmitic acid by of 250000 (250 K) primary enterocytes isolated from the duodenum or jejunum of Cpt1mt^fl/fl^ and iCPT1mt mice. Left two bars: Mice fed standard chow. Right two bars: Mice fed HFD for 3 days (n = 5–6, Unpaired t test, *P < 0.05, ***P < 0.001, ****P < 0.0001). (**B**) Oxygen consumption rate (OCR) of primary enterocytes per well, isolated from the (duodenum + jejunum) of Cpt1mt^fl/fl^ and iCPT1mt mice, incubated in KHB medium and subsequently injected with KHB medium or with an effective concentration of 5 mM glucose, 10 µg/mL Oligomycin (Oligo), 8 µmol/L FCCP and 5 µg/mL antimycin + 3.75 µmol/L rotenone (Anti + Rot). (**C**) Basal respiration, respiration linked to ATP synthesis, maximal respiration, proton leak and spare respiratory capacity of cells in B (n = 5–6, Unpaired t test, **P < 0.01, ***P < 0.001, ****P < 0.0001). (**A**) Enterocytes from 3 animals were pooled for each genotype (biological replicates) and the n values refer to technical replicates. (**B**,**C**) Each well represents a biological replicate of 1, while the n values refer to technical replicates. The data represent values from two independent experiments pooled together. (**A–C**) Data are presented as mean values ± SEM.

Primary enterocytes isolated from iCPT1mt mice fed standard chow showed an increased flux of palmitate oxidation in the duodenum and jejunum compared to Cpt1mt^fl/fl^ mice (*P* < 0.05) (Fig. 2A). When fed HFD for 3 days, primary enterocytes from the duodenum of iCPT1mt mice showed a lower rate of palmitate oxidation compared to Cpt1mt^fl/fl^ mice (*P* < 0.001), but the enterocytes from the jejunum of iCPT1mt mice showed an enhanced palmitate oxidation rate compared to controls (*P* < 0.0001) (Fig. 2A). In a mitochondrial stress test with the seahorse analyzer, primary enterocytes isolated from the duodenum and jejunum of iCPT1mt mice fed standard chow showed an increased basal and maximal (*P* < 0.0001) as well as increased respiration rate linked to ATP synthesis, proton leak (*P* < 0.001) and increased spare respiratory capacity (*P* < 0.01) (Fig. 2B,C) compared to primary enterocytes from Cpt1mt^fl/fl^ mice. The basal extracellular acidification rate (ECAR) of enterocytes from these chow-fed iCPT1mt mice was also higher than that of cells from Cpt1mt^fl/fl^ controls (*P* < 0.0001) (Sup Fig. 1A,B) as well as after the addition of oligomycin (*P* < 0.01) (Sup Fig. 1A,B). The addition of 2-deoxyglucose (2-DG), the competitive inhibitor of the enzyme hexokinase (HK1), abrogated the increased maximal respiration as well as the increase in ECAR seen after the addition of glucose, oligomycin and FCCP in these cells (Sup Fig. 1C). Enterocytes from iCPT1mt mice fed standard chow diet have an increased rate of glycolysis (*P* < 0.05), glycolytic capacity (*P* < 0.05) and glycolytic reserve (*P* < 0.05) (Sup Fig. 1C,D).

### Intestinal CPT1mt expression reduces visceral fat with HFD feeding without affecting body weight gain

**Figure 3:**
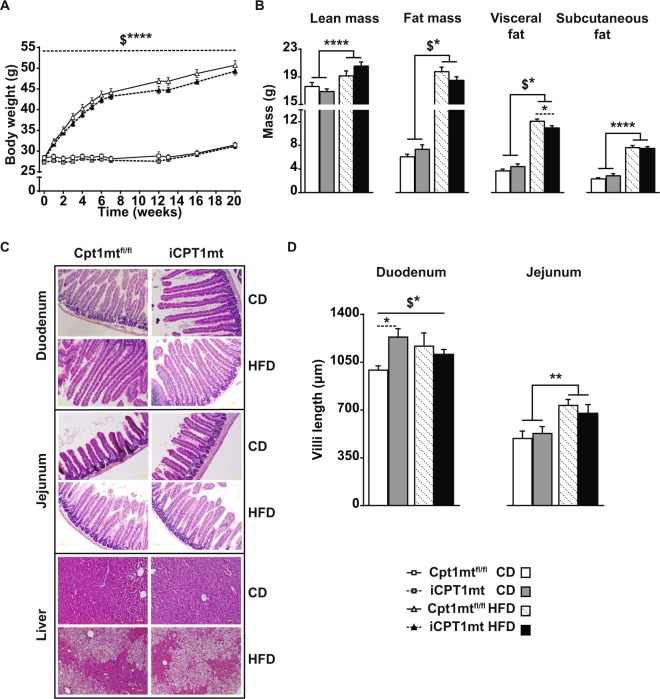
Intestinal CPT1mt expression reduces visceral fat with HFD feeding without affecting body weight gain. (**A**) Body weights of Cpt1mt^fl/fl^ and iCPT1mt mice on CD or HFD monitored over time (n = 11–17; Mixed- repeated measures (RM) ANOVA (time x diet x genotype), ****P < 0.0001 for interaction effects of diet x genotype ($)). (**B**) CT scan analyses of Cpt1mt^fl/fl^ and iCPT1mt mice fed CD or HFD for 19 weeks (n = 5–10; 2 × 2 factorial ANOVA (diet x genotype). *P < 0.05, ****P < 0.0001 for main effect of diet and post hoc tests (dashed lines) and interaction effects of diet x genotype ($). (**C**) Representative bright field microscopy images of H&E stained duodenum, jejunum and liver sections of Cpt1mt^fl/fl^ and iCPT1mt mice fed CD or HFD for 20 weeks at 20X magnification. The yellow bar represents a 100 μm scale. (**D**) Measurements of the villi lengths of Cpt1mt^fl/fl^ and iCPT1mt mice from H&E stained sections. Left: Duodenum, Right: Jejunum (n = 3–5; 2 × 2 factorial ANOVA (diet x genotype). *P < 0.05, **P < 0.01 for main effect of diet and post hoc tests (dashed lines) and interaction effects of diet x genotype ($)). (**A**,**B**,**D**) Data are presented as mean values ± SEM.

HFD feeding increased body weight (significant after 1 week, *P* < 0.0001), lean body mass (*P* < 0.0001) and subcutaneous fat mass (*P* < 0.0001) (microcomputed tomography (CT) measurement after 19 weeks on the diets) compared to CD feeding in all mice irrespective of genotype (Fig. 3A,B). Total fat mass and visceral fat mass showed an interaction effect (*P* < 0.05, diet x genotype for both) such that iCPT1mt mice had less visceral fat than Cpt1mt^fl/fl^ mice when fed HFD for 19 weeks (*P* < 0.05) (Fig. 3B).

A morphological analysis of the small intestine of iCPT1mt and Cpt1mt^fl/fl^ mice fed CD or HFD revealed an interaction effect in the villi lengths of their duodenum (*P* < 0.05, diet x genotype), such that iCPT1mt mice on CD had longer duodenal villi compared to Cpt1mt^fl/fl^ mice (*P* < 0.05) (Fig. 3C,D). HFD feeding led to an increase in the length of villi in the jejunum of all mice (*P* < 0.01) compared to CD feeding, with no genotype effects (Fig. 3C,D). The livers of these mice showed no genotype differences, but all mice on HFD, irrespective of genotype, showed markers of hepatic steatosis compared to CD fed mice (Fig. 3C).

### iCPT1mt mice and Cpt1mt^fl/fl^ control mice show similar energy intake and whole body energy metabolism when fed CD and when switched to HFD

**Figure 4:**
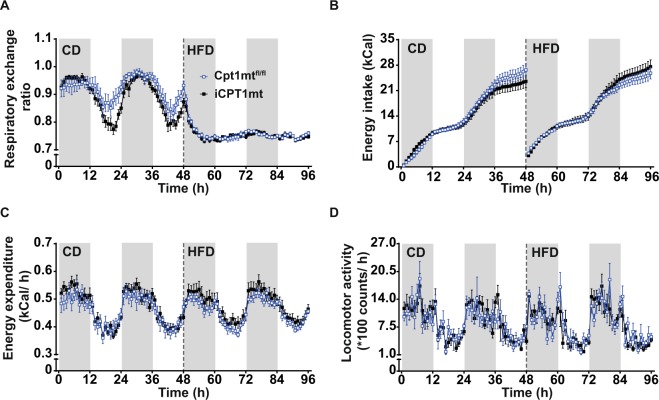
iCPT1mt mice and Cpt1mt^fl/fl^ control mice show similar energy intake and whole body energy metabolism when fed CD and when switched to HFD. (**A**–**D**) Indirect calorimetry data of Cpt1mt^fl/fl^ and iCPT1mt mice on CD for 48 h and HFD for the next 48 h. The grey and white bars in the background represent the periods of dark and light phases respectively. The dashed lines indicate when the mice were switched from CD to HFD feeding. (**A**) Respiratory exchange ratio as mean values of 1 h bins. (**B**) Cumulative energy intake in 1 h bins. (**C**) Energy expenditure (EE) as mean values of 1 h bins. (**D**) Locomotor activity as mean values of 1 h bins. (n = 8–11, Mixed RM ANOVA (diet × time × genotype, repeated measures for time and diet for **A,B** and **D**; ANCOVA for mean values per day with BW as the covariate for C). (**A**–**D**) Data are presented as mean values ± SEM.

Both iCPT1mt and Cpt1mt^fl/fl^ mice showed a similar decrease in the respiratory exchange ratio (RER) (*P* < 0.0001) when switched from CD to HFD feeding (Fig. 4A). No differences were observed in daily energy intake (Fig. 4B), energy expenditure (Fig. 4C) or locomotor activity (Fig. 4D) between the genotypes or on either of the two diets.

### Intestinal CPT1mt expression compromises glycemic control on the CD, but improves it over time on HFD

**Figure 5:**
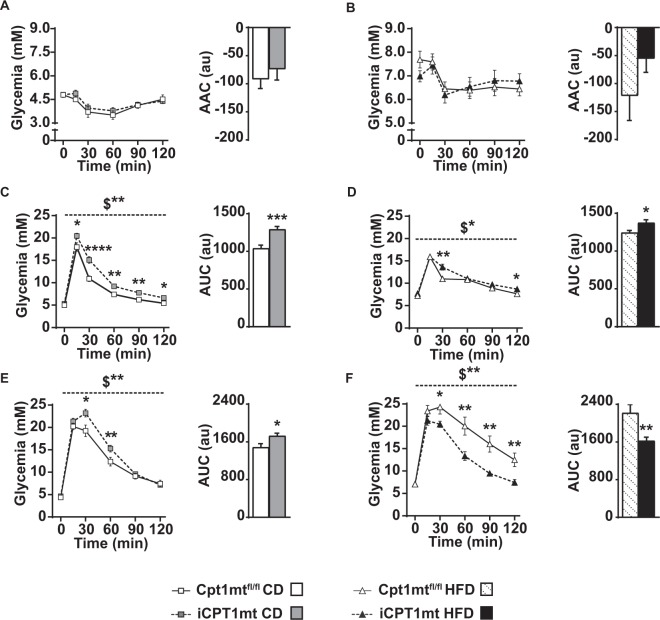
Intestinal CPT1mt expression compromises glycemic control on the CD, but improves it over time on HFD. (**A**,**B**) Insulin sensitivity test (IST) for Cpt1mt^fl/fl^ and iCPT1mt mice on CD and HFD, respectively. (**A**) Left: Tail blood glucose values for CD-fed mice at the time points indicated (n = 10–13). (**B**) Left: Tail blood glucose values for HFD-fed mice at the time points indicated. Right: Area above the curve (AAC) for the data on the left (n = 16–17). (C-D) Oral glucose tolerance test (OGTT) for Cpt1mt^fl/fl^ and iCPT1mt on CD and HFD respectively. (**C**) Left: Tail blood glucose values for CD-fed mice at the time points indicated. Right: Area under the curve (AUC) for the data on the left (n = 11–15). (**D**) Left: Tail blood glucose values for HFD-fed mice at the time points indicated. Right: Area under the curve (AUC) for the data on the left (n = 11–15). (**E**–**F**) Intra-peritoneal glucose tolerance test (IPGTT) for Cpt1mt^fl/fl^ and iCPT1mt on CD and HFD respectively. (**E**) Left: Tail blood glucose values for CD-fed mice at the time points indicated. Right: Area under the curve (AUC) for the data on the left (n = 11–15). (**F**) Left: Tail blood glucose values for HFD-fed mice at the time points indicated. Right: Area under the curve (AUC) for the data on the left (n = 11–15). (**A**–**F**) Mixed RM ANOVA (time x genotype) with repeated measures for time for the line graphs and unpaired t test for the bar graphs. *P < 0.05, **P < 0.05, ***P < 0.001, ****P < 0.0001 for main effect of genotype and simple effects analysis of genotype at each time point in the case of a significant interaction between time and genotype ($). Data are presented as mean values ± SEM.

An insulin sensitivity test (IST) on iCPT1mt and Cpt1mt^fl/fl^ control mice fed CD or HFD for 12 weeks did not reveal any differences between the two genotypes (Fig. 5A). Both HFD-fed iCPT1mt and Cpt1mt^fl/fl^ mice had developed IR as indicated by the increased baseline glucose levels (Fig. 5B). An oral glucose tolerance test (OGTT) after 13 weeks on the diets revealed that iCPT1mt mice on CD had impaired glucose clearance compared to Cpt1mt^fl/fl^ control mice (*P* < 0.01, time x genotype) (Fig. 5C). A pairwise comparison at each time point showed that iCPT1mt mice had higher circulating glucose levels compared to Cpt1mt^fl/fl^ mice, at every time point measured post glucose gavage (at t = 15 and 120 min, *P* < 0.05; at t = 30, *P* < 0.0001; at t = 60 and 90 min, *P* < 0.01). With HFD feeding, iCPT1mt mice also showed impaired glucose tolerance compared to Cpt1mt^fl/fl^ mice, though with a smaller difference between the two genotypes (Fig. 5D, time x genotype, *P* < 0.05), with iCPT1mt mice showing higher circulating glucose levels at 30 min (*P* < 0.01) and 120 min (*P* < 0.05) post glucose gavage.

After 16 weeks on the diets, mice fed CD continued to show a similar difference, with iCPT1mt mice exhibiting higher levels of circulating glucose in an intraperitoneal GTT (IPGTT) (*P* < 0.01, time x genotype) (Fig. 5E), specifically at 30 (*P* < 0.05) and 60 min (*P* < 0.01) post IP glucose injection. HFD-fed iCPT1mt mice, however, showed a reversal of glucose tolerance in the IPGTT. iCPT1mt mice cleared the IP-injected glucose better than Cpt1mt^fl/fl^ mice (*P* < 0.01, time x genotype) (Fig. 5F), with lower glycemia at 30 (*P* < 0.05), 60 (*P* < 0.01), 90 (*P* < 0.01) and 120 min (*P* < 0.01) post glucose injection.

### iCPT1mt mice have reduced circulating non-esterified fatty acid (NEFA) levels compared to Cpt1mt^fl/fl^ control mice

**Figure 6:**
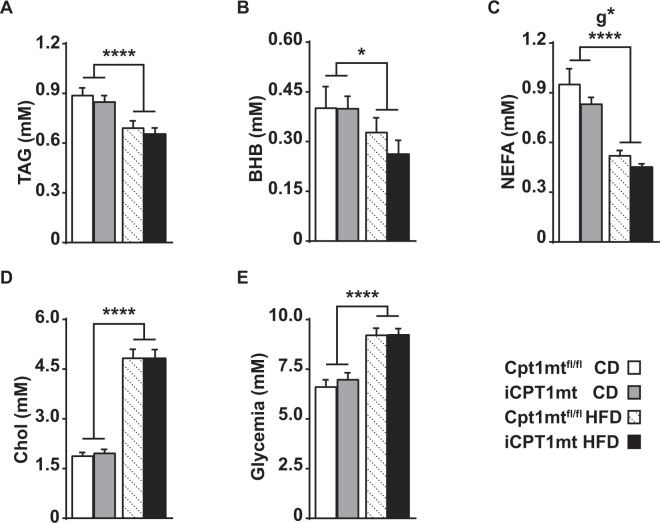
iCPT1mt mice have reduced circulating non-esterified fatty acid (NEFA) levels compared to Cpt1mt^fl/fl^ control mice. (**A**–**E**) Post prandial trunk blood plasma metabolite levels of Cpt1mt^fl/fl^ and iCPT1mt mice on CD and HFD. (**A**) Triacylglycerol (TAG). (**B**) β-hydroxybutyrate (BHB). (**C**) Non-esterified fatty acids (NEFA). (**D**) Cholesterol (Chol). (**E**) Glucose. (**A**–**E**) n = 11–17, 2 × 2 factorial ANOVA (diet x genotype). *P < 0.05, ****P < 0.0001 for main effect of diet and genotype (g). (**A**–**E**) Data are presented as mean values ± SEM.

An analysis of circulating post-prandial plasma metabolites after 20 weeks on CD or HFD revealed a main effect of diet such that all HFD-fed mice showed lower levels of circulating triacylglycerol (TAG) (*P* < 0.0001), β-hydroxybutyrate (BHB) (*P* < 0.05) and NEFA (*P* < 0.0001) than mice on CD (Fig. 6A–C). Plasma NEFA levels also showed a main effect of genotype (*P* < 0.05), but the *post hoc* tests did not reveal a significant difference between the genotypes on either diet (Fig. 6C). Plasma cholesterol as well as plasma glucose levels were higher in all HFD-fed mice than in CD-fed mice, irrespective of genotype (*P* < 0.0001) (Fig. 6D–E).

### iCPT1mt mice on CD, but not on HFD, show an upregulation of fatty acid synthesis, glycolysis and gluconeogenic genes in jejunal enterocytes compared to Cpt1mt^fl/fl^ mice

**Figure 7:**
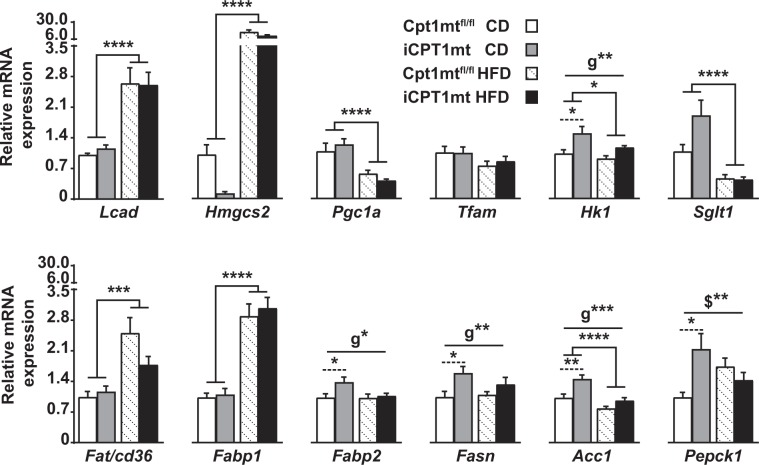
iCPT1mt mice on CD show an upregulation of fatty acid synthesis, glycolysis and gluconeogenic genes in jejunal enterocytes compared to Cpt1mt^fl/fl^ mice. Relative mRNA expression of genes in the jejunum of Cpt1mt^fl/fl^ and iCPT1mt mice fed CD or HFD for 20 weeks (n = 6–10, 2 × 2 factorial ANOVA (diet x genotype). *P < 0.05, **P < 0.001, ***P < 0.001, ****P < 0.0001 for main effects of diet or genotype (**g**) and post hoc tests (dashed lines) and interaction effects of diet x genotype ($). Data are presented as mean values ± SEM.

Gene expression analyses of the jejunal enterocytes of iCPT1mt and Cpt1mt^fl/fl^ mice after 20 weeks on CD and HFD revealed increased expression of genes involved in fatty acid uptake (*Fat/cd36*) (*P* < 0.001), fatty acid binding (*Fabp1*), FAO (*Lcad*), ketogenesis (*Hmgcs2*) (*P* < 0.0001) and malonyl-CoA synthesis (*Acc2*) (*P* < 0.0001) in all HFD-fed mice compared to CD-fed mice (Fig. 7, Sup Fig. 2 and Sup Table 1). The genes involved in glucose absorption through the apical enterocyte membrane (*Sglt1*) (*P* < 0.0001), glycolysis (*Hk1*) (*P* < 0.05) and *de novo* fatty acid synthesis (*Acc1*) (*P* < 0.0001) were downregulated in all HFD-fed mice compared to CD-fed mice (Fig. 7 and Sup Table 1). Genes involved in mitochondrial biogenesis were downregulated (*Pgc1a*) (*P* < 0.0001), or remained unchanged (*Tfam*) in all HFD-fed mice compared to CD-fed mice, irrespective of genotype. All iCPT1mt mice showed an upregulation of glycolysis (*Hk1*), fatty acid binding (*Fabp2*) (*P* < 0.05), *de novo* fatty acid synthesis (*Fasn* (*P* < 0.01) and *Acc1* (*P* < 0.001)) compared to Cpt1mt^fl/fl^ mice, and *post hoc* tests revealed that these effects were significant on the CD (*P* < 0.05 for *Hk1*, *Fabp2* and *Fasn*; *P* < 0.01 for *Acc1*). The gluconeogenic gene phosphoenolpyruvate carboxykinase 1 (*Pepck1*) showed an interaction effect in the jejunum (*P* < 0.01, diet x genotype) such that *Pepck1* was upregulated in the CD-fed iCPT1mt mice compared to Cpt1mt^fl/fl^ mice (*P* < 0.05), with no difference between the genotypes when fed HFD (Fig. 7, Sup Fig. 2 and Sup Table 1).

The duodenal enterocytes of all HFD-fed mice showed increased expression of *Lcad*, *Hmgcs2*, *Fat/cd36*, *Fabp1*, *Fabp2*, *Acc2* (*P* < 0.0001) and *Fasn* (*P* < 0.05) and reduced expression of *Pgc1a* (*P* < 0.0001) compared to CD-fed mice (Sup Fig. 2). The expressions of *Pgc1a* (*P* < 0.05) and *Acc2* (*P* < 0.01) were further downregulated in all iCPT1mt mice compared to Cpt1mt^fl/fl^ controls, and a *post hoc* test showed that this effect was significant for *Pgc1a* in iCPT1mt mice fed CD (*P* < 0.05) and for *Acc2* in iCPT1mt mice fed HFD (*P* < 0.01) (Sup Fig. 2). *Acc1* showed a genotype effect (*P* < 0.05), but the *post hoc* comparisons were not significant when compared within each diet (Sup Fig. 2). Expression of *Sglt1* showed an interaction effect (*P* < 0.01, diet x genotype) with its levels specifically upregulated in iCPT1mt mice fed CD (*P* < 0.01) (Sup Fig. 2).

The livers of iCPT1mt and Cpt1mt^fl/fl^ mainly showed an upregulation of all the genes tested in HFD-fed mice, compared to those fed CD (*P* < 0.05 for *Hmgcs2*, *P* < 0.01 for *Fasn* and *P* < 0.0001 for the other genes), except for *Pgc1a* and *Pepck1*, which showed no differences between the genotypes or diets (Sup Fig. 3). A western blot analysis of these tissue samples showed that protein level of the ketogenic marker HMGCS2 was downregulated in the duodenal and jejunal enterocytes but not in the liver of iCPT1mt mice on CD (*P* < 0.01 for duodenum, *P* < 0.05 for jejunum). HMGCS2 protein levels were also downregulated in the jejunal enterocytes (*P* < 0.05), but not in the duodenal enterocytes or in the liver, of HFD-fed iCPT1mt mice compared to Cpt1mt^fl/fl^ controls (Sup Fig. 4).

## Discussion

Studies using pharmacological approaches in rodents suggest that metabolic changes in the small intestine can reduce food intake and hence improve DIO^6,7^. Metabolic changes in the intestine may also contribute to the improvements in glucose homeostasis seen immediately after bariatric surgery in humans and rodents^4^. Here we report that enterocyte CPT1mt expression impaired glycemic control in CD-fed mice, but improved it in HFD-fed mice. These effects were independent of changes in body weight, but were accompanied by increased expressions of genes involved in fatty acid synthesis, glucose uptake, glycolysis and gluconeogenesis, especially in CD-fed iCPT1mt mice.

Using a previously established method, we isolated primary enterocytes from iCPT1mt and Cpt1mt^fl/fl^ mice^9,21^ and successfully processed and embedded them in a diluted matrigel matrix such that we could study their metabolic flux *in vitro*. The increased FAO flux in enterocytes isolated from chow-fed iCPT1mt mice compared to those from Cpt1mt^fl/fl^ mice is consistent with previous studies in which the CPT1mt protein was expressed in different tissues or cell types^12–17^. Together these results suggest that the enterocytes of iCPT1mt mice are capable of oxidizing more fatty acids than the enterocytes of control Cpt1mt^fl/fl^ mice when fed a low-fat diet. When fed HFD, the jejunal enterocytes of iCPT1mt can enhance their FAO capacity even further. A direct comparison is not possible between the FAO rates of enterocytes from chow and HFD fed mice because they were performed as independent experiments. Visually though, HFD feeding for 3 days seemed to increase the FAO flux of both duodenal and jejunal enterocytes of Cpt1mt^fl/fl^ mice and only the jejunal enterocytes of iCPT1mt mice. This is consistent with previous data from our lab, which showed that the gene expression of FAO enzymes was upregulated in the duodenum and jejunum of wildtype mice fed HFD for 3 days^23^. As for the decrease in the FAO rate in the duodenum of iCPT1mt mice compared to Cpt1mt^fl/fl^ mice, we speculate that the expression of CPT1mt protein may have maximized the FAO capacity of these cells, and they were not able to extend this capacity further at least after 3 days of HFD, but this remains to be tested.

The extracellular flux analysis revealed that pooled duodenal and jejunal enterocytes of chow-fed iCPT1mt mice had an overall increased basal metabolism, in the absence of any added substrate in the medium, compared to enterocytes from Cpt1mt^fl/fl^ mice. Providing glucose increased the rate of glycolysis and mitochondrial respiration in enterocytes from iCPT1mt mice, indicating that their glycolytic as well as oxidative respiration capacity were upregulated compared to enterocytes from Cpt1mt^fl/fl^ mice. It would be interesting to see how HFD feeding would affect these metabolic fluxes.

At the whole body level, we saw no differences in body weight gain between iCPT1mt and Cpt1mt^fl/fl^ mice when fed CD or HFD for up to 20 weeks. This is similar to what we observed in intestinal SIRT3 overexpressing (iSIRT3) mice^9^, suggesting that increasing FAO flux in the enterocytes of mice does not cause a change in body weight gain. A body composition analysis revealed, however, that HFD-fed obese iCPT1mt mice had less visceral fat than Cpt1mt^fl/fl^ mice. Both iCPT1mt and Cpt1mt^fl/fl^ control mice showed similar changes in postprandial circulating TAG, BHB, cholesterol and glucose levels when fed HFD vs CD. These effects were similar to those seen in iSIRT3 mice on HFD and CD, and were consistent with the species-specific effects observed in long-term HFD-fed mice^27,28^. We saw no genotype differences in energy intake or energy homeostasis when the mice were fed CD or switched to HFD. This was also similar to our findings in iSIRT3 mice^9^, indicating that a constitutive upregulation of FAO in the enterocytes does not affect energy intake or whole body energy metabolism. It would be interesting to see how the energy metabolism in these mice might change after feeding them CD or HFD for several weeks.

Both iCPT1mt and Cpt1mt^fl/fl^ control mice showed similar responses to exogenous insulin after 12 weeks of CD or HFD feeding. iCPT1mt mice however responded differently to exogenous glucose depending on the diet, the route of glucose administration and/or the duration of CD or HFD feeding. These differences could be related to the mode of glucose administration, but may also indicate that adult iCPT1mt mice that may have already been intolerant to glucose, slowly reversed this phenotype when fed HFD chronically. If so, it may explain why the iCPT1mt mice were still comparatively intolerant to exogenous glucose after 13 weeks on HFD, but had reversed this phenotype by 16 weeks. The idea of a biphasic response of glucose intolerance in HFD fed C57Bl6 mice seen after 3 days of HFD feeding and then between 12 to 16 weeks of HFD feeding fits with our idea of a gradual change in glucose tolerance^29^. The reduced visceral fat mass in HFD-fed iCPT1mt mice could also be involved in the improved glucose tolerance, since visceral fat is well known to be associated with the incidence of metabolic syndrome^30^. These results were partly similar and partly different from our findings in iSIRT3 mice. Enterocyte overexpression of SIRT3 had no effect in mice fed CD but did protect them from developing IR and glucose intolerance under conditions of DIO^9^.

Expressions of genes for fatty acid binding and fatty acid synthesis were upregulated specifically in iCPT1mt mice compared to Cpt1mt^fl/fl^ controls on CD. This could indicate that CPT1mt expression in the jejunal enterocytes led to increased *de novo* fatty acid synthesis in these cells, perhaps to funnel fatty acids into the mitochondria for the constitutively active FAO. This assumption is consistent with data from isolated rat hepatocytes expressing the CPT1mt protein. Even in the presence of high glucose and insulin that favor lipogenesis, these cells were able to oxidize *de novo* synthesized LCFA^12^.

Interestingly, the glycolytic gene *Hk1* was upregulated in the jejunal enterocytes of iCPT1mt mice fed CD. This is consistent with the seahorse data from isolated enterocytes, which showed that enterocytes from iCPT1mt mice had a higher rate of glycolysis than enterocytes from Cpt1mt^fl/fl^ mice when provided with glucose. Mitochondrial acetyl-CoA generated via FAO cannot directly leave the mitochondria and fuel fatty acid synthesis. It needs to enter the TCA cycle to generate citrate, which can then leave the mitochondria via the citrate shuttle to fuel *de novo* lipogenesis^31^. The pyruvate derived from enhanced glycolysis undergoes oxidative decarboxylation, and the resulting Acetyl-CoA also enters the mitochondria to fuel the TCA cycle. When TCA cycle intermediates become limiting, accumulating mitochondrial acetyl-CoA form ketone bodies. In the enterocytes of CD-fed iCPT1mt mice, however, the protein levels of ketogenic HMGCS2 were significantly downregulated. Therefore, we would favor the hypothesis that the accumulating acetyl-CoA could be funneled into the TCA cycle which would eventually fuel lipogenesis. Interestingly, the expression of the gluconeogenic gene *Pepck1* was also increased in the jejunal enterocytes of iCPT1mt mice, indicating that these cells were producing glucose. Together these results indicate that the jejunal enterocytes of CD-fed iCPT1mt mice might have developed futile cycles of FAO and lipogenesis, as well as glycolysis and gluconeogenesis.

Concomitantly, in the duodenal enterocytes of CD-fed iCPT1mt mice, we saw an increase in *Sglt1* expression. SGLT1 is the glucose transporter in the apical membrane that absorbs glucose from the intestinal lumen, thus supporting the idea of increased glucose absorption. The increased villi length in the duodenum of iCPT1mt mice fed CD as well as the higher levels of blood glucose seen in iCPT1mt mice, 15 minutes after oral, but not IP glucose bolus support the idea of increased glucose absorption from the intestine of iCPT1mt mice fed CD.

On HFD, all mice showed an overall increase in the expressions of genes related to fatty acid uptake, fatty acid binding, FAO and ketogenesis in their duodenal and jejunal enterocytes. Expressions of genes related to glucose absorption, glycolysis and gluconeogenesis were either downregulated in all mice or remained unchanged. Together these results indicate that the futile cycles generated in CD-fed iCPT1mt mice were absent in HFD-fed mice. How these enterocyte metabolic changes might affect whole body glucose homeostasis is still unclear.

In iSIRT3 mice fed HFD, increased ketogenesis in the small intestine was associated with the improved insulin sensitivity and glucose tolerance^9^. In iCPT1mt mice, however, HMGCS2 protein levels were significantly lower and circulating BHB levels were unchanged, indicating that these effects on improved glycemic control were unrelated to ketogenesis. We believe that the increased glucose absorption in the duodenum and the upregulated gluconeogenesis in the jejunum might contribute to the reduced glucose tolerance seen in iCPT1mt mice fed CD. Unlike hepatic gluconeogenesis that is inhibited by insulin, intestinal gluconeogenesis is known to be enhanced in the post absorptive period^32^. The enhanced intestinal gluconeogenesis supposedly provides more glucose for hepatic portal vein glucose sensors that may signal the brain to inhibit eating as reviewed in^33^. Perhaps the higher glucose levels observed in the OGTT and IPGTT are the result of substantially enhanced glucose absorption and/or gluconeogenesis in the enterocytes of iCPT1mt mice. Another possible explanation is that the permanently enhanced gluconeogenesis and glycolysis in the enterocytes of iCPT1mt mice fed CD limits their capacity to utilize large oral or intraperitoneal loads of glucose properly, leading to impaired glycemic control. Data from rodent models show that gluconeogenesis in the gut can contribute anywhere from 5 to 25% of whole body endogenous glucose production depending on the diet as reviewed in^34^. Mice with a liver specific or liver and intestinal specific knock out of the gluconeogenic gene glucose-6-phosphatase, catalytic subunit (*G6pc*) showed that in the absence of hepatic gluconeogenesis, intestinal gluconeogenesis is essential to maintain physiological levels of blood glycemia^35^. Together these data indicate that changes in enterocyte metabolism can contribute to and influence systemic glucose concentrations substantially, which supports our idea that enterocyte gluconeogenesis is capable of contributing to the glycemic dysregulation we see in iCPT1mt mice.

Perhaps on HFD, iCPT1mt mice have enough LCFA and carbohydrate intermediates to fuel the constitutive upregulation of FAO and the TCA cycle and therefore do not need a massive upregulation of glucose absorption or gluconeogenesis to fuel the *de novo* synthesis of fatty acids. The constitutive upregulation of FAO under these conditions could slowly reverse the adverse effects seen under CD fed conditions. It would be interesting to see whether iCPT1mt mice might be prevented from developing DIO and/or impaired glycemia if they were directly weaned onto the HFD. Consistent with this idea hepatic CPT1mt expression was able to prevent the development of DIO and IR in adult mice fed HFHS diet after the expression of CPT1mt using an adeno-associated virus^14^. Also both hepatic and skeletal muscle expressions of CPT1mt were able to reverse already established IR and impaired glycemia in DIO mouse models without affecting body weight^15,17^. In fact, these studies did not show any adverse effects of CPT1mt expression in mice fed standard chow. The main difference between these mouse models and our intestinal transgenic mice, other than the obvious difference in the organs we targeted, is that our mice express CPT1mt constitutively from embryonic development, whereas in these other models the CPT1mt expression was induced in adulthood. An inducible Villin-Cre model, in which we could specifically express CPT1mt in the mouse enterocytes in adulthood, might provide a clearer picture to whether an upregulation of enterocyte FAO would protect from the development of DIO and IR, without the negative effects seen on CD.

The declining slope in the OCR of the enterocytes seen in the mitochondrial stress test was most likely due to a decrease in cell viability over time. Previous studies with intestinal crypts, organoids or intestinal stem cells in the seahorse also showed similar declines in the OCR over time^36,37^. We believe that this was a drawback of embedding primary enterocytes in a 3D layer of diluted matrigel. We would like to emphasize, however, that the present study represents the first successful attempt to analyze the bioenergetics of live primary enterocytes from adult rodents. As epithelial cells, enterocytes are notoriously difficult to culture alive and especially as single cells. In our attempts to culture these cells, we performed several trial experiments with different cell surface attachment protocols, all of which led to steep declines in the OCR of the enterocytes. The method of embedding the cells in matrigel was the only one that worked and gave a consistently, but comparatively slowly and similarly decreasing slope in every experiment. We also tested different dilutions of matrigel to determine the best conditions under which we were able to achieve a consistent range of OCR as well as a consistent slope.

In sum, these results show that persistent changes in mouse enterocyte FAO do not influence daily energy intake or energy homeostasis and body weight, but affect whole body glucose homeostasis under different dietary conditions. Further studies should try to elucidate the exact mechanism of these effects and dissociate the contribution of the molecular and metabolic changes in the gut versus other organs.

## Electronic supplementary material

Supplementary information
