# Supplementary material for: Enhancing enterocyte fatty acid oxidation in mice affects glycemic control depending on dietary fat
Source: Sci Rep. 2018 Jul 17;8:10818. doi: 10.1038/s41598-018-29139-6 (PMC6050244; doi:10.1038/s41598-018-29139-6)
Supplement: Supplementary file 1 — Supplementary information [file 41598_2018_29139_MOESM1_ESM.pdf]

Enhancing enterocyte fatty acid oxidation in mice affects glycemic control depending on dietary fat

Deepti Ramachandran[1], Rosmarie Clara[1], Shahana Fedele[1], Ladina Michel[1], Johannes Burkard[1], Sharon Kaufman[1], Abdiel Alvarado Diaz[2], Nadja Weissfeld[1], Katrien De Bock[2], Carina Prip-Buus[3,4,5], Wolfgang Langhans[1], Abdelhak Mansouri[1,*]

[1]Physiology and Behavior Laboratory, ETH Zurich, Schwerzenbach, Switzerland

[2]Excercise and Health Laboratory, ETH Zurich, Schwerzenbach, Switzerland

[3]Inserm, U1016, Institut Cochin, Paris, France

[4]CNRS, UMR 8104, Paris, France

[5]Université Paris Descartes, Sorbonne Paris Cité, Paris, France

[*]**Corresponding author:** Physiology and Behavior Laboratory, Institute of Food, Nutrition and Health, Schorenstrasse 16, 8603 Schwerzenbach, Switzerland.

Tel: +41-44-655-7485; Fax: +41-44-655-7206.

E-Mail: abdelhak-mansouri@ethz.ch

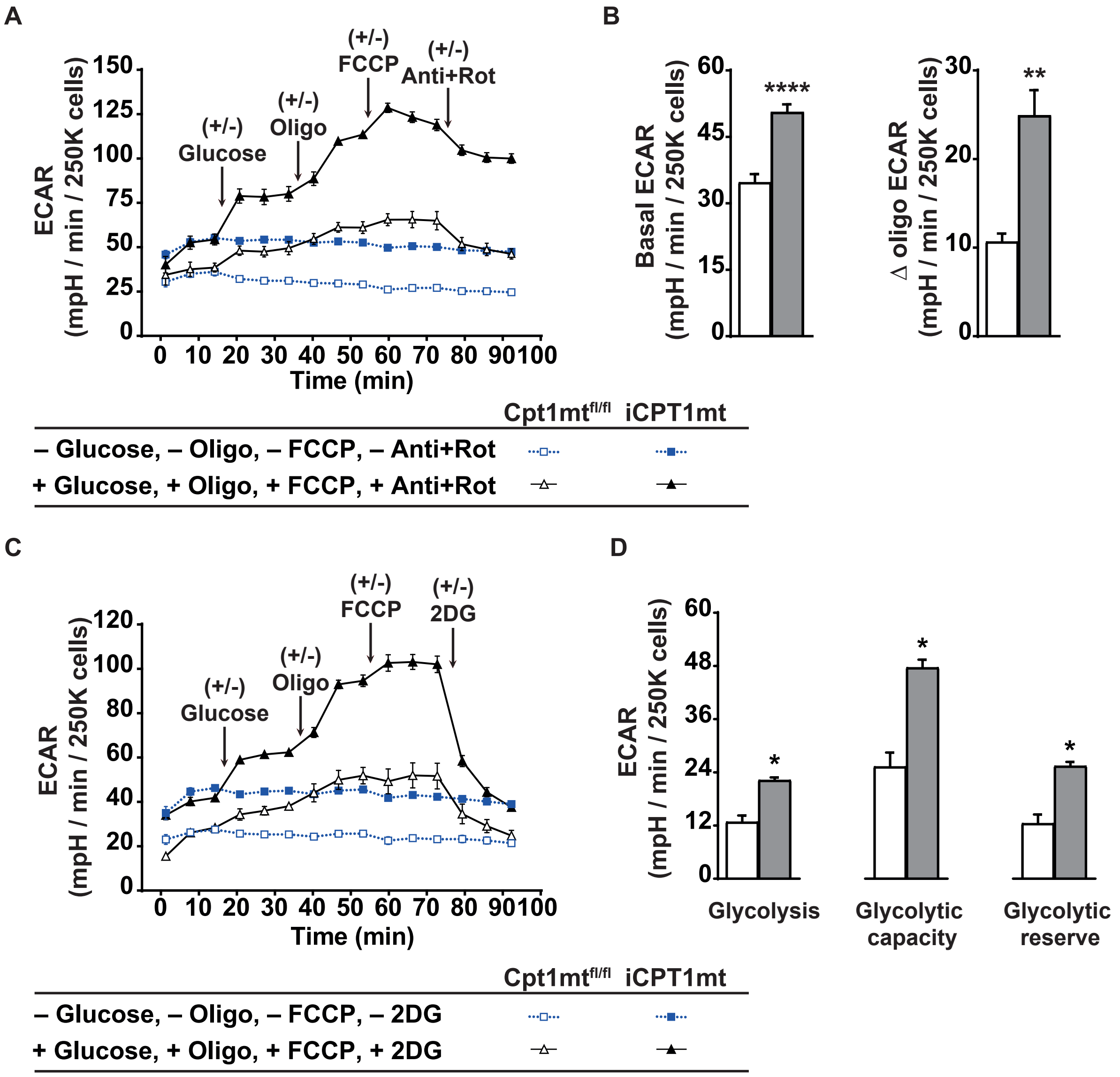

Supplementary figure 1: Primary enterocytes from iCPT1mt mice fed standard chow show increased glycolysis and glycolytic reserve compared to Cpt1mtfl/fl control mice.
(A) Extra cellular acidification rate (ECAR) of 250000 (250K) primary enterocytes per well, isolated from the small intestine (duodenum + jejunum) of Cpt1mt$^{fl/fl}$ and iCPT1mt mice, incubated in KHB medium and subsequently injected with KHB medium (squares) or with an effective concentration of 5mM glucose, 10 μg/mL Oligomycin (Oligo), 8 μmol/L FCCP and 5 μg/mL antimycin + 3.75 μmol/L rotenone (Anti+Rot) (triangles). (B) Left: Basal ECAR of cells in (A) before the addition of glucose with values for the same genotype pooled (n = 18-19). Right: The change in ECAR (Δ ECAR) in (A) induced by oligo (n = 5-6). (C) Extra cellular acidification rate (ECAR) of 250K primary enterocytes isolated from the small intestine (duodenum + jejunum) of Cpt1mt$^{fl/fl}$ and iCPT1mt mice incubated in KHB medium and subsequently injected with KHB medium (squares) or with an effective concentration of 5mM glucose, 10 μg/mL Oligomycin (Oligo), 8 μmol/L FCCP and 100mM 2-deoxyglucose (2-DG) (triangles). (D) Glycolysis, glycolytic capacity and glycolytic reserve calculated from the values in (C) (n = 3-5). (B) Unpaired t test and (D) Mann-Whitney test. $^*P < 0.05$, $^{**}P < 0.01$, $^{****}P < 0.0001$. Data are presented as mean values ± SEM.

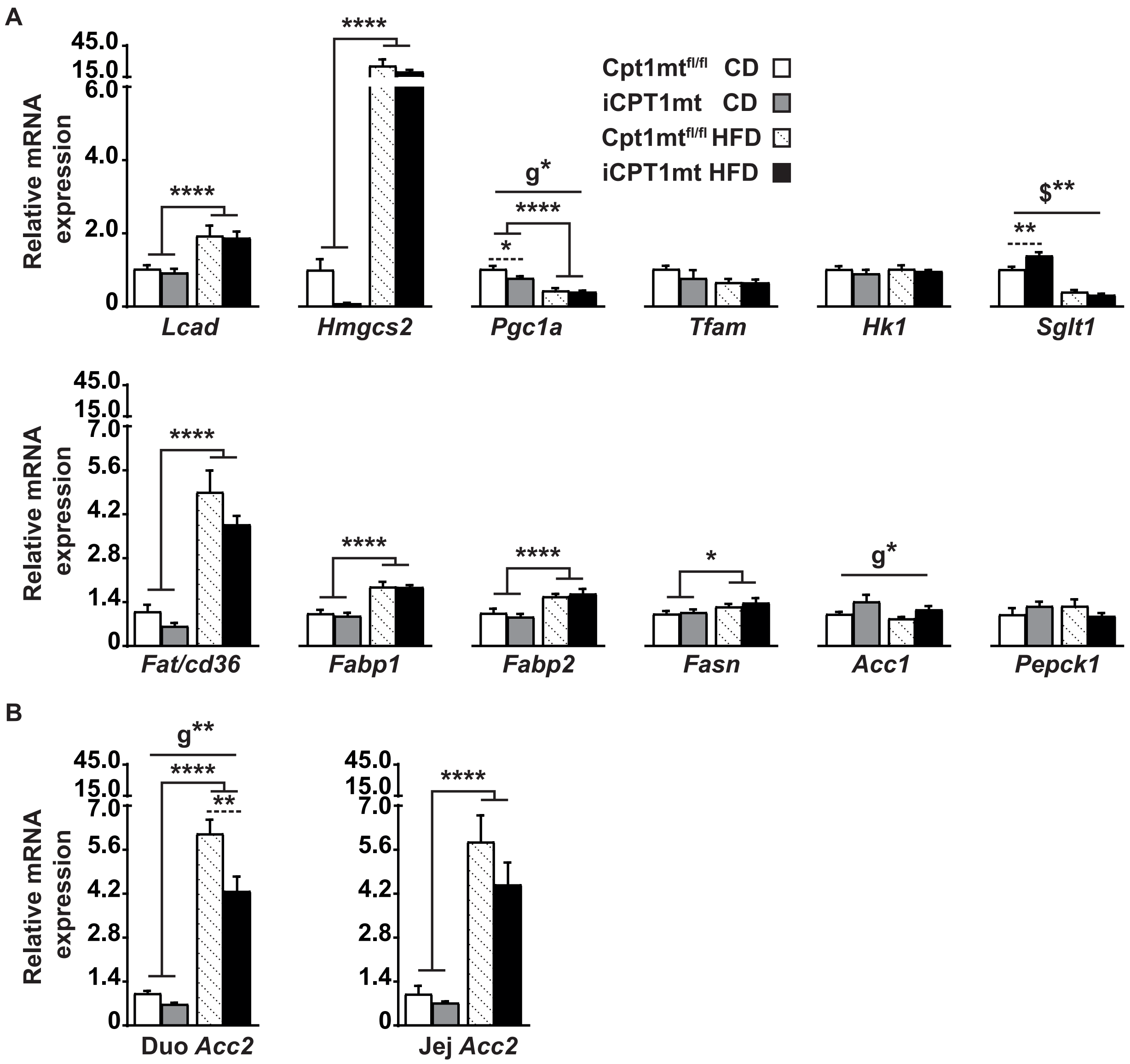

Supplementary figure 2: iCPT1mt mice on CD show an upregulation of glucose uptake in the duodenal enterocytes compared to Cpt1mtfl/fl mice.

(A) Relative mRNA expression of genes in the duodenum of Cpt1mt$^{fl/fl}$ and iCPT1mt mice fed CD or HFD for 20 weeks. (B) Relative mRNA expression of Acc2 in the duodenum (duo) and jejunum (jej) of Cpt1mtfl/fl and iCPT1mt mice fed CD or HFD for 20 weeks. (n = 6-10, 2 x 2 factorial ANOVA (diet x genotype). *P < 0.05, **P < 0.001, ***P < 0.001, ****P < 0.0001 for main effects of diet or genotype (g) and post hoc tests (dashed lines) and interaction effects of diet x genotype ($). Data are presented as mean values ± SEM.

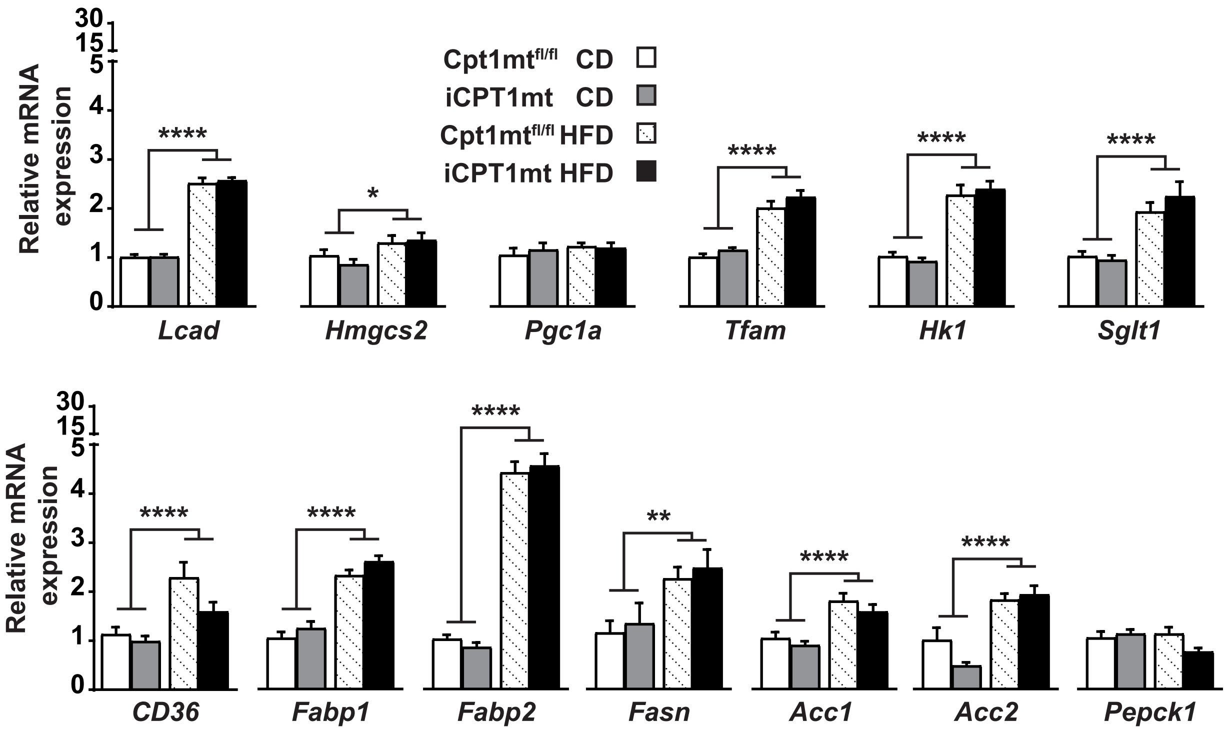

Supplementary figure 3: Livers of iCPT1mt mice showed no gene expression differences from Cpt1mt$^{fl/fl}$ control mice when fed CD or HFD.

Relative mRNA expression of genes in the liver of Cpt1mt$^{fl/fl}$ and iCPT1mt mice fed CD or HFD for 20 weeks. (n = 6-10, 2 x 2 factorial ANOVA (diet x genotype). *P < 0.05, **P < 0.001, ***P < 0.001, ****P < 0.0001 for main effects of diet or genotype (g) and post hoc tests (dashed lines) and interaction effects of diet x genotype ($). Data are presented as mean values ± SEM.

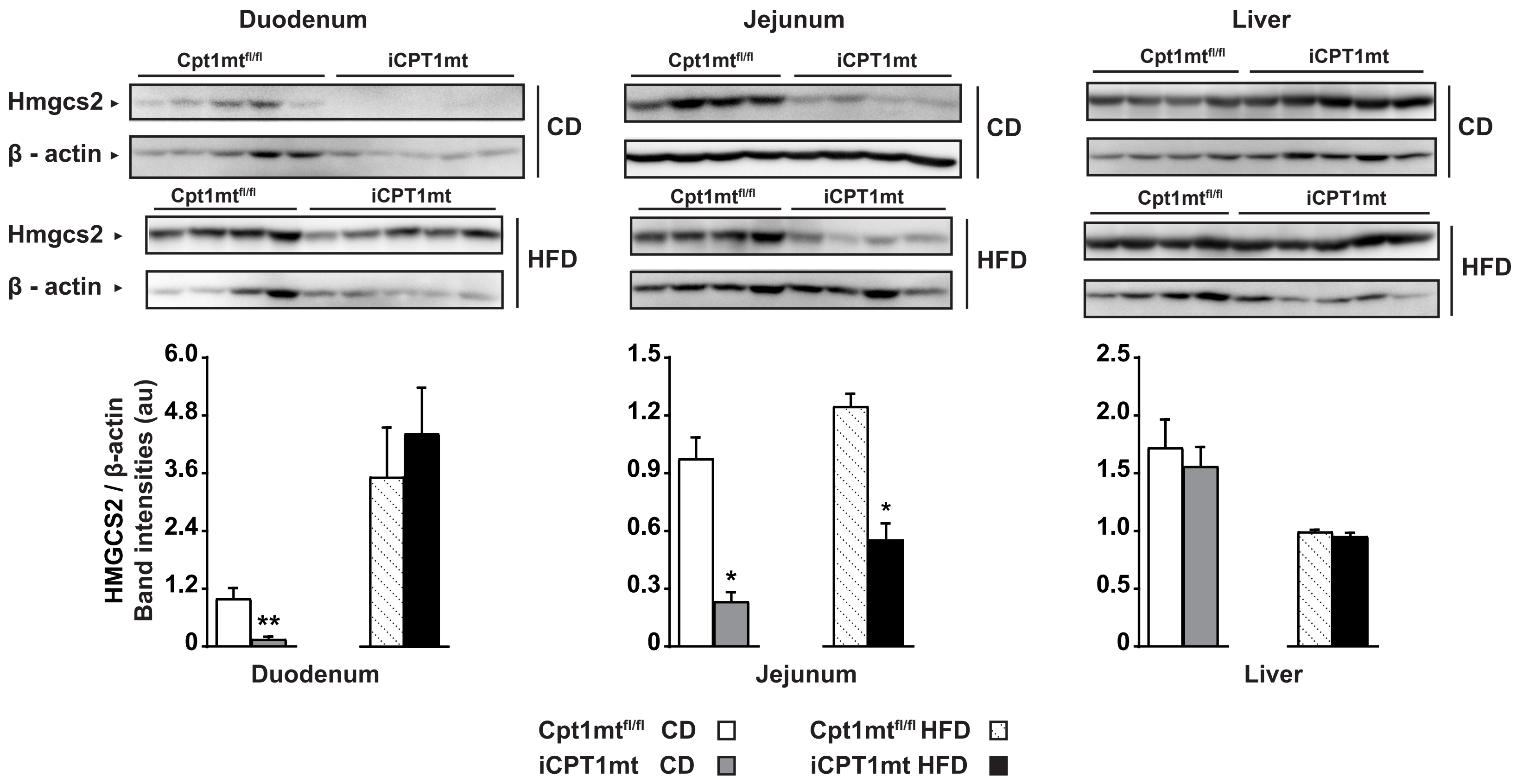

Supplementary figure 4: iCPT1mt mice show reduced HMGCS2 protein expression in the duodenum and jejunum.

Western blot analysis for 3-Hydroxy-3-Methylglutaryl-CoA Synthase 2, (HMGCS2) and β-actin protein expression from tissue samples of Cpt1mt$^{fl/fl}$ and iCPT1mt mice fed control diet (CD) or high-fat diet (HFD) for 20 weeks. (A) Representative pictures of western blot bands from the duodenum, jejunum and liver. The dotted line separates discontinuous lanes from the same gel. (B) Quantification of band intensities. (n = 5, Unpaired t test; **P < 0.01 for Duodenum CD. n = 4-5 , Mann-Whitney test; *P < 0.05 for Jejunum CD and HFD and Liver CD and HFD). Data are presented as mean values ± SEM.

Supplementary Table 1: Primers for RTqPCR analyses.

Gene name	Primers
Long chain acyl-CoA dehydrogenase (*Lcad*)	f: 5' gtctggactccggttctgc 3'
	r: 5' ccgtggagttgcacacatt 3'
3-Hydroxy-3-methylglutaryl-CoA synthase 2 (*Hmgcs2*)	f: 5' agataccaccaacgcctgtt 3'
	r: 5' aatgtcaccacagaccacca 3'
Peroxisome proliferative activated receptor gamma, coactivator 1 alpha (*Pgc1a*)	f: 5' ttctcgacacaggtcgtgtt 3'
	r: 5' gtgtgcggtgtctgtagtgg 3'
Transcription factor A, mitochondrial (*Tfam*)	f: 5' aaggatgattcggctcagg 3'
	r: 5' ggctttgagacctaactgg 3'
Hexokinase 1 (*Hk1*)	f: 5' tgtgggtcacgatgtagcc 3'
	r: 5' ccacatccaggtcaaattcc 3'
Sodium-dependent glucose co-transporter (*Sglt1*)	f: 5' aagagcgaatcgacctgga 3'
	r: 5' gaagcatcctttcttcttctgg 3'
Fatty acid translocase (*Fat/cd36*)	f: 5' ttgaaaagtctcggacattgag 3'
	r: 5' tcagatccgaacacagcgta 3'
Fatty acid binding protein 1, liver (*Fabp1*)	f: 5' aagtggtccgcaatgagttc 3'
	r: 5' cttccagcttgacgactgc 3'
Fatty acid binding protein 2, intestinal (*Fabp2*)	f: 5' acggaacggagctcactg 3'
	r: 5' ttaccagaaacctctcggaca 3'
Fatty acid synthase (*Fasn*)	f: 5' gctgctgttggaagtcagc 3'
	r: 5' agtgttcgttcctcggagtg 3'
Acetyl-CoA carboxylase alpha (Acaca or *Acc1*)	f: 5' cctgaagaccttaaagccaatgc 3'
	r: 5' ccagcccacactgcttgta 3'
Acetyl-Coenzyme A carboxylase beta (Acacb or Acc2)	f: 5' gattcccagtttgggcact 3'
	r: 5' ccttcaaagccactaccatgt 3'
Phosphoenolpyruvate carboxykinase 1 (*Pepck1*)	f: 5' ggagtacccattgagggtatcat 3'
	r: 5' gctgagggcttcatagacaag 3'
Peptidylprolyl Isomerase B (*Ppib*)	f: 5’ ttcttcataaccacagtcaagacc 3’
	r: 5’ accttccgtaccacatccat-3’
